# The kinetics of multibranch integration on the dendritic arbor of CA1 pyramidal neurons

**DOI:** 10.3389/fncel.2014.00127

**Published:** 2014-05-13

**Authors:** Sunggu Yang, Valentina Emiliani, Cha-Min Tang

**Affiliations:** ^1^Department of Neurology, University of Maryland School of Medicine, BaltimoreMD, USA; ^2^Wavefront-engineering Microscopy Group, Neurophotonics Laboratory, CNRS UMR 8250, Paris Descartes UniversityParis, France; ^3^Baltimore Veterans Adminstration Medical Center, BaltimoreMD, USA

**Keywords:** multibranch integration, 3D digital holography

## Abstract

The process by which synaptic inputs separated in time and space are integrated by the dendritic arbor to produce a sequence of action potentials is among the most fundamental signal transformations that takes place within the central nervous system. Some aspects of this complex process, such as integration at the level of individual dendritic branches, have been extensively studied. But other aspects, such as how inputs from multiple branches are combined, and the kinetics of that integration have not been systematically examined. Using a 3D digital holographic photolysis technique to overcome the challenges posed by the complexities of the 3D anatomy of the dendritic arbor of CA1 pyramidal neurons for conventional photolysis, we show that integration on a single dendrite is fundamentally different from that on multiple dendrites. Multibranch integration occurring at oblique and basal dendrites allows somatic action potential firing of the cell to faithfully follow the driving stimuli over a significantly wider frequency range than what is possible with single branch integration. However, multibranch integration requires greater input strength to drive the somatic action potentials. This tradeoff between sensitivity and temporal precision may explain the puzzling report of the predominance of multibranch, rather than single branch, integration from in vivo recordings during presentation of visual stimuli.

## Introduction

Individual thin dendritic branches are fundamental functional units in the nervous system (Branco and Hausser, 2011). Experimental data support the concept that they can operate as quasi-independent processing and signaling units capable of non-linear behavior (Mel, 1993; Wei et al., 2001). In combination with their parent dendritic branches, these thin distal dendrites can function in two distinct modes (Gasparini and Magee, 2006; Katz et al., 2009). If distributed synaptic inputs arrive on multiple distal branches, the depolarization on each branch may be below the threshold for recruiting local active conductances in a regenerative manner and yet be sufficient to trigger a somatic sodium spike. This is sometimes referred to as the traditional “integrate and fire” model (Abbott, 1999), the “synaptic democracy” model (Yuste, 2011), and the “global” model of integration. Alternatively, if synaptic inputs arrive in a clustered pattern on a single or a few distal dendrites, the focused inputs could initiate a non-linear response on the distal dendrite which is then relayed to and summed linearly in the more proximal compartment. This is referred to as either the “two-layer” or the “compartmentalization” model of integration (Mel, 1993; Golding and Spruston, 1998; Häusser and Mel, 2003; Poirazi et al., 2003a, b; Polsky et al., 2004; Larkum and Nevian, 2008; Winnubst and Lohmann, 2012). The advantages of the two-layer model of integration are well understood. It is more efficient in evoking somatic action potentials and can do so at the lowest synaptic strengths. By placing amplification close to the input optimal signal-to-noise performance can be achieved. This is analogous to the mechanism in a two-stage electronic amplification system, such as the preamplifier-power amplifier system typically used in electrophysiology; the amplification is entirely carried out in the preamplifier. Two-stage integration can also greatly increase the computational power of the neuron over that of global integration because it increases the number of non-linear operations that a single neuron can possess (Mel, 1993; Häusser and Mel, 2003). In the two-stage mode of integration it is the dendritic branch, rather than the synapse, that is the elementary unit of signaling. The tradeoffs associated with these two modes of integration have not been adequately examined, and doing so may lend insights into the controversy regarding the functional impacts of global and two-layer integration.

Whether integration on pyramidal neuron follows a global or a two-stage model is difficult to address because it depends on the spatial-temporal pattern of the inputs which is an in vivo phenomenon that may change with different physiological stimuli. Investigators from Konnerth’s group addressed this challenge using calcium imaging in an in vivo study of pyramidal neurons in the visual cortex in response to directionally selective visual inputs (Jia et al., 2010). They found that orientation-selective synaptic inputs were widely distributed throughout the dendritic field rather than being clustered on individual dendrites. This finding is more consistent with the global model of integration. The extent to which this conclusion can be applied to other pyramidal neurons in response to different physiological stimuli is not known. For example, Takahashi et al. (2012) reported that in layer 2/3 pyramidal neurons of the barrel cortex, related inputs frequently arrived synchronously on neighboring synapses, creating the possibility of local non-linear integration, which is more consistent with the two-stage model (Takahashi et al., 2012). In this study we take another approach towards addressing the global vs. two-stage controversy. We compared the property of single- and multibranch integration in response to photolysis as a surrogate for two-stage and global integration, respectively. The critical parameters differentiating the global and two-stage mode of integration are the precise location of non-linear integration within the dendritic arbor and the active conductances expressed at that location. Precise photolytic stimulation with complex 3D digital holographic-generated patterns allowed us to systematically examine this issue. Single- and multibranch integration was used to simulate clustered and distributed synaptic inputs that have different thresholds for non-linear integration. We found that the dendritic arbor of CA1 pyramidal neurons can support both the global and the two-stage modes of integration. The global mode of integration is less sensitive to low strength stimuli, but allows for accurate response following over a greater frequency range, while the two-stage mode of integration has high sensitivity but allows for accurate response following only at low frequencies.

## Materials and methods

### Brain slice preparation

**Figure 1:**
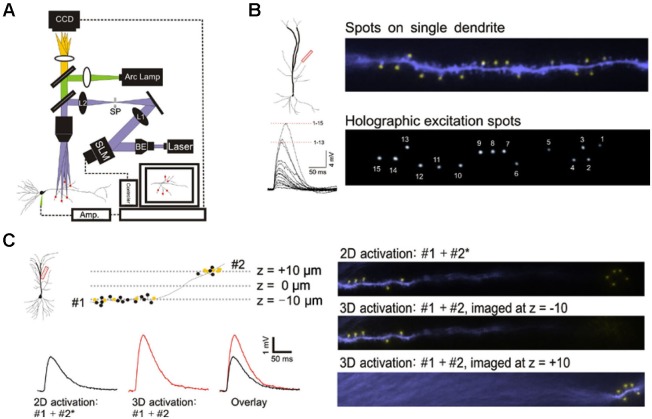
**3D digital holographic photolysis of oblique spines. (A)** Schematic of the 3D digital holographic setup. The locations of the sites to be stimulated are first identified and their 3D coordinates determined from fluorescence imaging of the dendrite. An in-house algorithm is then used to generate a digital holographic pattern of those coordinates which is project by a phase modulating spatial light modulator (SLM). The output of a 150 mW, 405 nm diode laser is expanded by a beam expander (BE) to fill the aperture of the SLM. The beam is then telescoped by two lenses (L1 and L2) to fill the back aperture of the microscope objective. A spatial filter (SF) is used to block the zero-order beam of the hologram from reaching the specimen. **(B)** Simultaneous photostimulation at multiple spines. The distal oblique of CA1 neuron was photostimulated. The yellow spot indicate sites of simultaneous uncaging on multiple spines (*upper panel*). Spot size is similar to that of spine (*compare with lower panel*). The spatially summed response (*lower left*) demonstrate non linear integration on single oblique dendrite.** (C)** Simultaneous photostimulation in two planes. Dendritic spines located at two imaging planes (#1 and #2) along a single dendrite are targeted. The yellow spots represent spines that are stimulated and the black spots represent those that are not stimulated. 2D stimulation of #1 and #2* (x,y-coordinates of #2 but with the z-coordinate set at -10) produces a lower amplitude (black trace) compared to simultaneous 3D activation (#1 + #2) (red trace).

**Figure 2:**
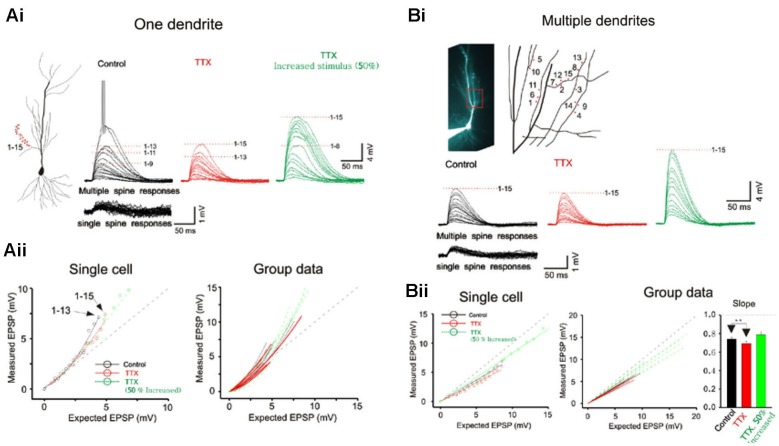
**Integration on spines. (A)** The photostimulation is distributed over 15 spines along a single dendrite without TTX. The somatic voltage responses to stimulation of individual spines (lower panels) and the responses to increasing number of simultaneous spines (upper panels) are shown in *black*. In presence of TTX, the responses are shown in *red*. The responses to increased laser stimulation are shown in *green*. The cell responses are plotted to examine the linearity of summation (expected vs. measured voltage response). Group data is obtained without TTX (*n* = 7), with TTX (*n* = 5) and with increased stimulation in TTX (*n* = 5). Supralinear summation is observed with single branch integration. **(B)** Photostimulation for the 15 spines along multiple branches. Two or four spines are stimulated per dendrite. The responses of the cell are plotted to examine the linearity of summation (expected vs. measured voltage response). Sublinear summation is observed with multibranch integration. Group data is obtained without TTX (*n* = 7), with TTX (*n* = 7) and with increased stimulation in TTX (*n* = 7). Error bars represent SE. ** *p* < 0.05.

**Figure 3:**
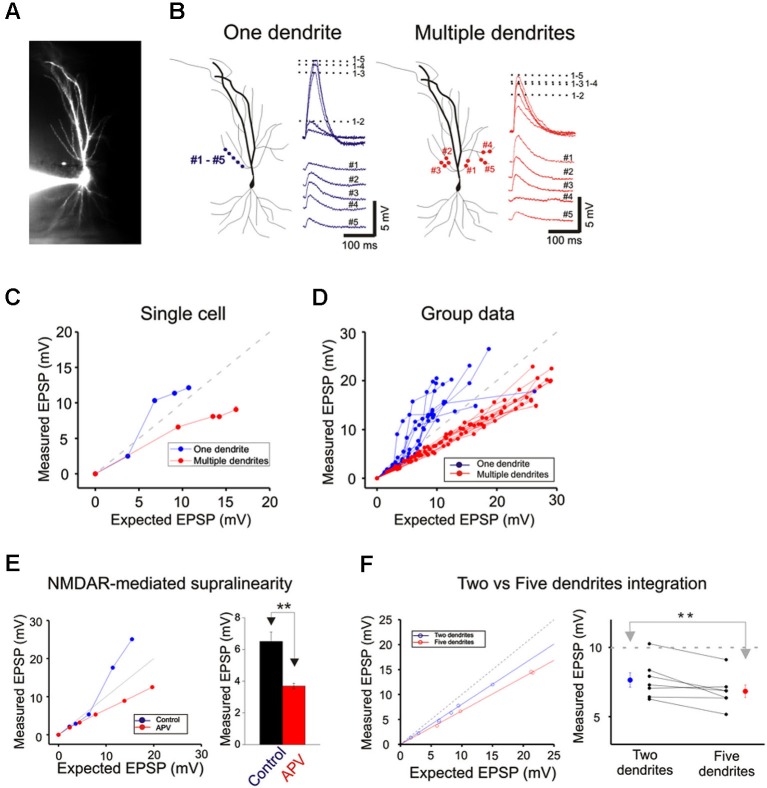
**Integration on a single dendrite is fundamentally different from that on multiple dendrites. (A)** Wide field image of a CA1 pyramidal neuron filled with Alexa 594. **(B)** Locations of focal photolysis of caged glutamate directed on a single oblique dendrite are labeled in blue. The stimulation is distributed over 5–7 spots spread out over an 80–100 micron length of an individual dendrite. Locations of photolysis for the multibranch stimulation are directed on five separate oblique dendrites in the same cell (labeled in red). Two sites located in the mid dendritic region are stimulated per dendrite. The somatic voltage responses to stimulation of individual spots or dendrites are shown in the lower panels. The responses to an increasing number of simultaneous spots or dendrites are shown in the upper panels. For example the trace labeled 1–5 in the one dendrite panel represents the response to simultaneous stimulation of all five blue spots.** (C)** The responses of the cell shown in panel (B) are plotted to examine the linearity of summation (expected vs. measured voltage response). Sublinear summation is observed with multibranch integration, whereas an abrupt transition to supralinear summation is observed when the expected voltage reached ∼5 mV with single dendrite summation. **(D)** The finding shown in **C** was obtained from 13 recordings of single dendrites and 14 recordings from multiple dendrites. The average slope of the multibranch summation is 0.68.** (E)** NMDAR-mediated dendritic spikes. The supralinear dendritic spike (*n* = 5, 6.5 ± 0.6) is blocked by APV (100 µ; *n* = 5; 3.7 ± 0.17, *p* < 0.05, paired *t*-test). Error bars represent SE. ** *p* < 0.05.** (F)** Magnitude of sublinear multi-branch integration increases with the number of branches. The linearity of summation between two branches and five branches is compared within the same cell (left). The measured EPSP for an expected EPSP of 10 mV is plotted for 7 cells (right). Error bars represent SE. ** *p* < 0.05.

**Figure 4:**
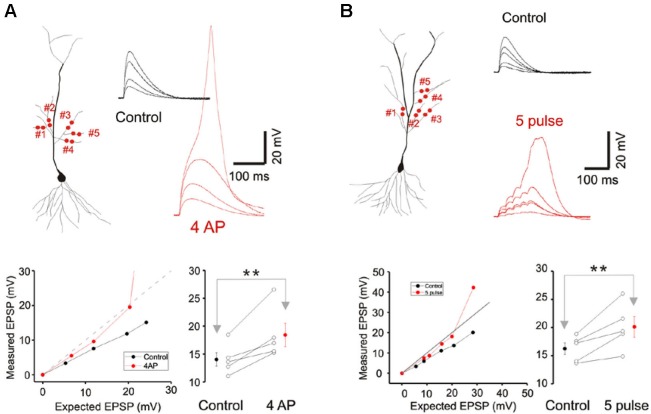
**A-type potassium conductance contributes to sublinear integration. (A)** Blockade of the A-type potassium conductance by the addition of 4AP removes the sublinear multi-branch summation at the oblique dendrites. It also uncovers the latent NMDA conductance and lowers the threshold for calcium spikes. Group data (*n* = 5) for the observed EPSP to a stimulus with an expected EPSP of 20 mV is shown to the lower right. Error bars represent SE. ** *p* < 0.05. **(B)** Sublinear multi-branch integration is abolished during burst stimulation (50 Hz). Instantaneous multibranch summation (black traces) is compared with summation to a burst of inputs (red traces). Summation is less sublinear with the burst stimulus. Group data (*n* = 5) for the observed EPSP to a stimulus with an expected EPSP of 25 mV is to the lower right. Error bars represent SE. ** *p* < 0.05.

All procedures were approved by the Institutional Animal Care and Use Committee at the University of Maryland School of Medicine. Sprague-Dawley rats (postnatal age: 3–6 weeks) were deeply anesthetized with halothane. The brains were quickly removed and placed into chilled (4°C), oxygenated (5% CO_2_ and 95% O_2_) slicing medium containing (in mM): 4 KCl, 1.23 NaH_2_PO_4_, 10 MgSO_4_, 0.5 CaCl2, 26 NaHCO_3_, 10 glucose, and 212.7 sucrose. Hippocampal slices (300 mm thickness) were cut using a vibrating tissue slicer and transferred to a holding chamber containing oxygenated physiological saline that contained (in mM): 124 NaCl, 4 KCl, 1.23 NaH_2_PO_4_, 1.5 MgCl_2_, 2.5 CaCl_2_, 26 NaHCO_3_, and 10 glucose. Individual slices were then transferred to a recording chamber and oxygenated physiological saline was continuously superfused at a rate of 0.7 ml/min. Certain experiments were carried out at 32°C (those illustrated in Figures 1, 2, 3, 4, 7, and 8) and the remaining experiments at room temperature.

**Figure 5:**
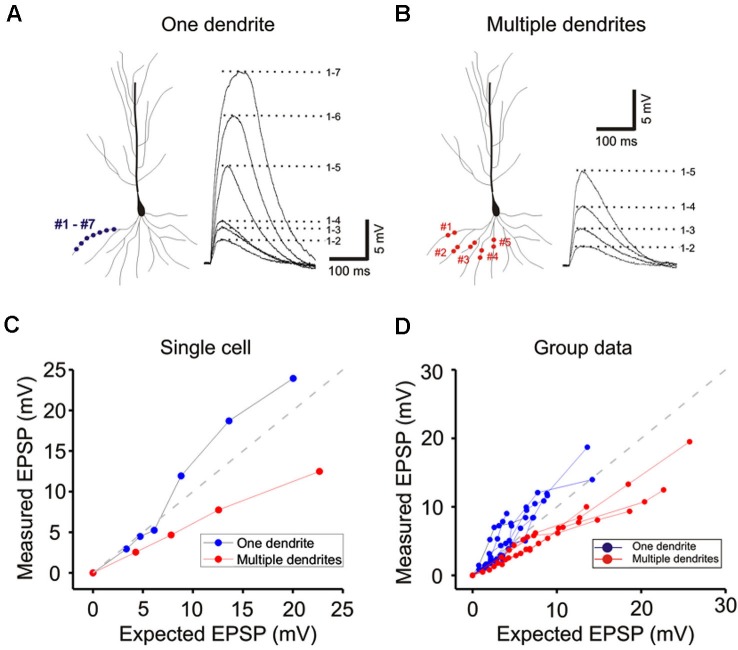
**Basal dendrites integration is qualitatively similar to integration on oblique dendrites. (A)** Response of a single basal dendrite to progressive increasing strength of stimulation distributed over 7 sites. **(B)** Response of the same cell to stimulation on five separate branches. **(C)** Responses shown in A and B are plotted and compared for linearity. **(D)** Group data from 8 cells.

**Figure 6:**
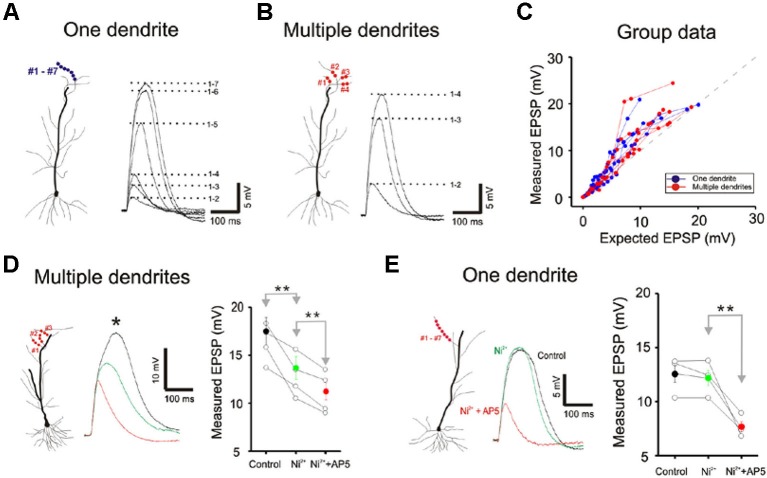
**Little difference is apparent between single and multibranch summation in the distal apical tuft. (A)** Response of a single distal apical dendrite to progressively increasing number of stimulation spots. **(B)** Response of the same cell to stimulation on multiple dendritic branches**. (C)** Group data from 8 cells. Responses to very strong stimulation levels are not displayed. **(D)** Conductances that contribute to non-linear multibranch integration of tuft. Nickel sensitive calcium conductances contribute partially to the supralinear summation at the multiple tuft dendrites. The remaining component is eliminated with application of AP5. Error bars represent SE. ** *p* < 0.05. **(E)** Conductances that contribute to non-linear single branch integration of tuft. Blockade of the T/R-type calcium channel by the addition of nickel does not affect the supralinear summation at the single tuft dendrites. However, NMDA receptor blocker, AP5 (100 µ) completely eliminate the supralinearity. Error bars represent SE. ** *p* < 0.05.

**Figure 7:**
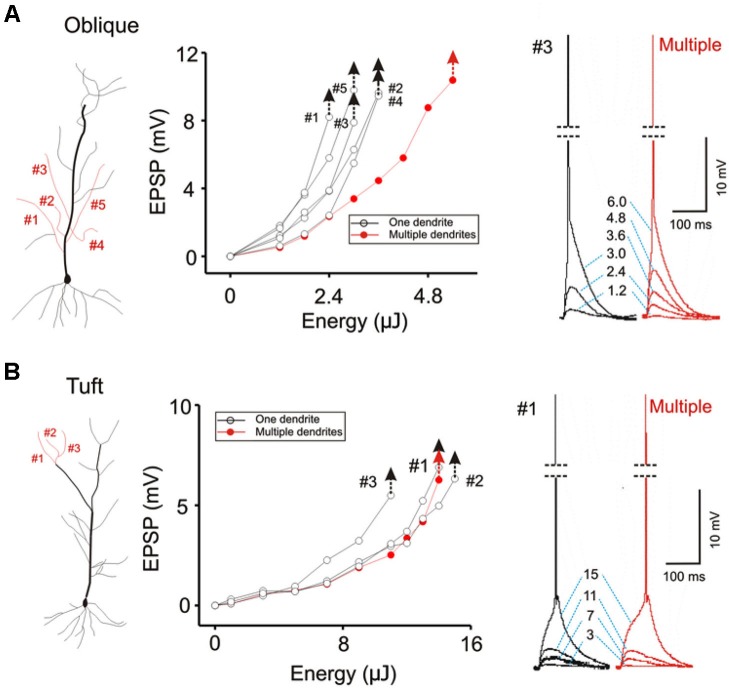
**Contrasting modes of integration translates into different thresholds for eliciting action potentials. (A)** The threshold for evoking somatic action potential is compared for five oblique dendrites when they are stimulated individually (black hollow circles) and simultaneously (solid red circles). Individual responses for each stimulus intensity are shown on the right. The threshold for single-branch stimulation is about half of that for multi-branch stimulation. The latency between stimulation and the spike is brief. **(B)** The same procedure is repeated for apical tuft dendrites. The threshold for single- and multi-branch stimulation is not significantly different at the tuft. The latency between stimulation and spike is slower.

**Figure 8:**
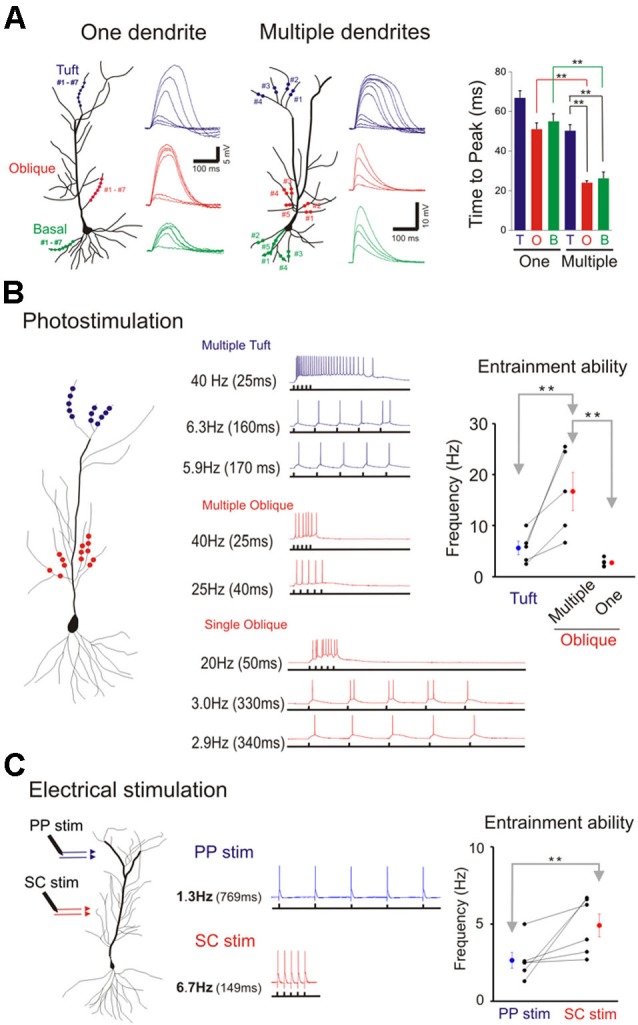
**Domain specific response kinetics. (A)** Differential response kinetics between single- and multi-branch integration and between different dendritic domains. In the presence of TTX time to peak is significantly slower for single branch integration compared to that for multibranch integration of oblique and basal dendrites (right panel). Error bars represent SE. ** *p* < 0.05. Time to peak is also significantly slow for multibranch integration of the tuft compared to that of oblique and basal dendrites. **(B)** Entrainment properties of tuft vs. oblique dendrites and single vs. multiple dendrites. The five stimuli were given at different frequencies. The highest frequency at which the somatic action potential can still precisely follow the dendritic input (entrainment) is recorded. Multibranch oblique integration had faster entrainment ability than either single branch oblique or multibranch tuft integration. Error bars represent SE. ** *p* < 0.05. **(C)** The differential frequency response between PP (blue) and SC (red) pathway. The highest frequency at which the somatic action potential can still precisely follow the dendritic input (entrainment) is recorded. SC pathway had better entrainment ability than PP pathway. Error bars represent SE. ** *p* < 0.05.

### Brain slice recording

Whole-cell patch recordings were obtained using an Axon instruments Axoclamp 700B Amplifier (Molecular Devices), and pClamp Version 10.2 software was used for data acquisition. Recording pipettes had tip resistances of 3–7 MΩ when filled with a solution containing (in mM): 135 K-gluconate, 5 KCl, 1 MgCl_2_, 0.02 CaCl_2_, 0.2 EGTA, 10 HEPES, 4 Na_2_-ATP, and 0.3 Na-GTP. The pH and osmolarity of intracellular solution were adjusted to 7.3 and 290 mOsm, respectively. Alexa 594 (50 µM) was included in the internal solution for visualization of dendrites. Recordings were done in “current-clamp” configuration and cells were held at −65 mV. For electrical stimulation experiment, synaptic responses were evoked with 15–60 µA, 0.4 ms current pulses delivered through a concentric bipolar stimulating electrode (FHC, 100 µm o.d.).

### 3D digital holography

The procedures for digital holographic photolysis were explained in detail in an earlier methods paper (Lutz et al., 2008; Yang et al., 2011). Briefly, the holographic beam was brought into the optical axis of an upright fluorescence microscope (Olympus BX51) below the epi-fluorescence unit, with a longpass dichroic mirror (Figure 1). The output beam of a 150 mW, 405 nm diode laser (CNI Laser) is expanded by a beam expander (BE) (3X) to fill the short axis of a reflective spatial light modulator (SLM) (LCOS Hamamatsu, model X10468-05). The SLM plane is projected onto the back aperture of the microscope objective through a telescope (L1, *f*_1_ = 500 mm; L2, *f*_2_ = 200 mm). The magnification of the telescope is chosen in order to match the SLM short axis with the diameter of the objective’s back aperture (Olympus, 60x, W 0.9NA). The undiffracted component of the hologram (zero order spot) is removed by placing a small (<0.5 mm) anodized metal plate on antireflective coated glass plate at the focal plane of L1 (spatial filter (SF)). This plane is conjugate to the image plane of the microscope. In order to change the total number of spots of excitation without changing the intensity of the remaining spots, spots that were not needed for excitation were steered onto the same small SF for blocking the zero order beam. The algorithm for the phase hologram calculation and calibration of the temporal spatial resolution were previously described (Yang et al., 2011).

### Pharmacological agents

Concentrated stock solutions of various pharmacological agents were initially prepared and diluted in physiological saline to a final concentration before use. For uncaging experiments, MNI-caged-L-glutamate (Tocris, Ellisville, MO) or and MNI-L-glutamate trifluoro acetate (Femtonics, Hungary) were prepared each day at final concentration in physiological solution. All agonists and antagonists were purchased from Sigma (St. Louis, MO) or Tocris (Ellisville, MO). The presence or absence of tetrodotoxin (TTX) is provided for each experiment.

## Results

### 3D digital holographic photolysis

This study was made possible by 3D digital holographic photolysis (Anselmi et al., 2011; Yang et al., 2011; Go et al., 2012), therefore it is useful to describe its strengths and potential weaknesses. A schematic of the optical system is illustrated in Figure 1A. 3D digital holography has three characteristics important to experimental investigation of dendritic integration: (1) the ability to efficiently deliver light to diffraction limited spots and photorelease glutamate in a way that mimics normal synaptic transmission (Nikolenko et al., 2008; Yang et al., 2011); (2) the ability to stimulate simultaneously; at a large number of locations in arbitrary, user defined, temporal-spatial patterns, (Figure 1B); and (3) the ability to stimulate in 3D space such as is traversed by multiple dendrites oriented in different directions (Figure 1C).

The holographic system used in this study utilizes single photon excitation, whereas other studies have utilized two-photon excitation. It is reasonable to question whether the novel observations of this study could be accounted for by differences in the ability of single- and two-photon photolytic methods to focus the light. This issue was addressed by three independent methods: direct visualization of the illumination pattern and spot size on the target dendrite in the hippocampal slice, comparison of the kinetics of the photolytic responses produced by single- and two-photon holographic photolysis at individual target sites, and comparison of responses to branch wide stimulation with single- and two-photon photolysis. If no significant differences could be observed under these three conditions, there would be little reason to expect greater light scattering of single photon excitation to account for unexpected findings associated with multibranch integration. Figure 1B shows the area of illumination when the holographic pattern was directed on a dendrite that had been dialyzed with an intracellular fluorescent dye. The spatial resolution under experimental conditions is consistent with the previously measured optical resolution for this system of 0.4 and 2 µm in the transverse and axial directions, respectively (Yang et al., 2011). This degree of spatial resolution is not unexpected when studying those structures <60 µm from the slice surface. We had previously shown that the kinetics of the voltage clamped holographically induced response are comparable to a fast EPSC (Yang et al., 2011). Most importantly, the single branch response to single-photon holographic stimulation (Figures 1B, 2A, 3C, 3D, 4C, 4D) is indistinguishable from what has been reported for two-photon stimulation (Branco and Hausser, 2011). Since multibranch integration is the sum of single branch integration, anomalous behavior of multibranch integration should not be dismissed as artifacts of single photon excitation.

## Differences between single- and multibranch integration

Whole cell patch recordings were made on CA1 neurons in transverse hippocampal slices. Alexa594 was placed in the patch electrode and was allowed to dialyze into the dendritic arbor. Once the fluorescence signal of the oblique dendrites became visible, the 3D coordinates of the photolysis sites on the spines were identified. Individual spots first stimulated individual spines (lower panels Figure 2A) and were then sequentially combined (upper panels). In the presence of TTX the supralinearity of single branch integration was decreased (red vs. black exponentially fitted lines in Figure 2A). The average depolarization immediately before the onset of the sodium spike occurred was about 7.8 mV at an expected response of 5.3 mV (group data in Figure 2Aii). The non-linearity for this group data was 1.47 in the absence of TTX (*n* = 7) and 1.27 with TTX (*n* = 5; *p* > 0.05, *t*-test). The responses of single oblique dendrites to holographic photolysis are nearly identical to that previously reported for synaptic stimulation (Polsky et al., 2004) and 2P photostimulation (Branco and Hausser, 2011). After validating the reliability of the stimulating technique at the level of individual spines, the same holographic technique was then employed to stimulate five separate oblique dendrites. The responses in this case were significantly sublinear (Figure 2B). The slope of multibranch sublinearity was 0.74 ± 0.02 (*n* = 7) without TTX (black traces) and was 0.69 ± 0.03 (*n* = 7) with TTX (red traces). Voltage-gated sodium channels have a relatively small but statistically significant influences on the sublinearity of multibranch integration (*p* > 0.05, paired *t*-test). Such contrasting tendencies of single vs. multibranch integration were not altered by increasing stimulating power to activate spine and shaft together (Figure 2 *green*).

Next, photolysis was directed at 5 to 7 spots distributed over ∼100 µm length of the mid portion of a single oblique dendrite and TTX (1 µM) was added to permit observation of synaptic responses over a wider range of intensity (Figures 3A and left panel, 3B). Integration was linear at low stimulus intensities (<3–5 mV), supralinear at moderate intensities (3–10 mV), and trending towards saturation at high intensities ( >5–10 mV) (Figures 3C and D). The supralinear integration is NMDAR-mediated (Figure 3E).

The same procedures were then repeated for multibranch stimulation in the same cells. Stimuli delivered to each of the five dendrites were distributed between at least two separate spots of photolysis in the mid-dendritic region (right panel, Figure 3B). The average degree of linearity observed here from 14 cells is 0.68 (Figures 3C and D), was substantially more sublinear than previously revealed from summation of two branches (Cash and Yuste, 1999; Polsky et al., 2004). The group data also show that multibranch integration is also more sublinear than single branch integration over the same stimulus intensity range (Figure 3D). The nearly identical behavior illustrated in Figures 2 and 3 also suggests that spine stimulation does not influence the fundamental nature of single- and multibranch integration.

We examined the effect of varying the number of branches involved in integration between two and five in the same cell. The degree of sublinearity lessened with a decrease in numbers of branches that were involved (Figure 3F). The measured EPSP for an expected EPSP of 10 mV is 7.65 ± 0.52 mV for two branches and 6.84 ± 0.45 mV for five branches (*n* = 7, *p* < 0.05, paired-*t* test). These findings suggest that multibranch integration may recruit additional conductances in addition to those involved in single branch integration, and that these are expressed near the locus where the branches converge. With increasing numbers of stimulated branches, these conductances are recruited at a rate that exceeds the more linearly summated depolarizing signal.

Cash and Yuste (1999) had suggested that the A-type potassium conductance (I_A_) could act as a counterbalancing force to the recruitment of NMDA conductance during branch point summation and linearize integration. We investigated whether I_A_ serves a more prominent role in multibranch integration. Indeed, applying the A-type potassium channel antagonist, 4AP (3 mM), reversed the sublinearity (Figure 4A; Control: 14.04 ± 1.23 mV, 4AP: 18.43 ± 2.09 mV, *n* = 5, *p* < 0.05, paired *t*-test). To circumvent the poor target selectivity of 4AP, we examined whether sublinear summation could be attenuated during a burst stimulus, a condition that promotes inactivation of I_A_. Indeed, sublinear summation is eliminated during burst stimulation (Figure 4B; Control: 16.25 ± 1.03 mV, burst: 20.12 ± 1.82 mV, *n* = 5, *p* < 0.05, paired *t*-test). Taken together, these findings suggest that the difference between single- and multibranch integration of the oblique dendrites could be accounted for by the recruitment of additional A-type potassium conductance at the locus where distal oblique branches converge.

We next examined single- and multibranch summation on the basal dendrites. Qualitatively, single- and multibranch summation of the basal dendrites was similar to that of the oblique dendrites (Figure 5). The opposing tendencies of single- and multibranch summation of basal dendrites suggest that basal dendrites are functionally similar to oblique dendrites.

### Domain specific multibranch integration

We next compared single- and multibranch integration in the distal apical tuft dendrites to test the idea that the mode of integration can vary in different domains of the dendritic arbor. Because the tuft receive inputs that are distinct from those of oblique and basal dendrites, it would not be surprising to find domain-specific differences. Integration on individual tuft dendrites, as was in the case of oblique dendrites, is linear at weak intensities, followed by supralinear summation at moderate intensities, and finally trending to sublinear summation at high intensities (*Blue* Figures 6A, B and C). But in contrast to the case at the oblique dendrites, multibranch integration of the tuft is not sublinear at low or moderate stimulation intensities (*red* Figures 6A, B and C). The base of the apical tuft where signals from distal tuft dendrites converge and pass through to reach the soma, is thought to express high levels of voltage gated calcium conductances (Larkum et al., 1999, 2009). In support of this proposed mechanism, we found the application of nickel (50–100 µM) which preferentially blocks the T/R-type calcium channels, partially attenuated the multibranch supralinear summation at the apical tuft (Figure 6D; Control: 17.50 ± 1.43 mV, Ni^2+^: 13.68 ± 1.19 mV, *n* = 5, *p* < 0.05, paired *t*-test). The remainder of the supralinear component could be eliminated with application of AP5 (100 µM Ni^2+^ + AP5: 11.28 ± 0.87 mV, *n* = 6, *p* < 0.05, paired *t*-test with Ni^2+^ group). In contrast, the supralinear summation of single branches in the tuft was not blocked by Ni^2+^ (Figure 6E; Control: 12.59 ± 0.61 mV, Ni^2+^: 12.28 ± 0.56 mV, *n* = 5, *p* < 0.05, paired *t*-test) but it was blocked by AP5 (100 µM Ni^2+^ + AP5: 7.66 ± 0.35 mV, *n* = 5, *p* < 0.05, paired *t*-test with Ni^2+^ group).

The focus of most studies on dendritic integration has been on the linearity of integration and the efficiency with which inputs can generate a somatic action potential. Efficiency is defined in terms of the amount of synaptic excitation required to produce a somatic action potential. The two-layer model of integration achieves greater efficiency by allowing supralinear summation to occur on the distal dendritic compartment. We confirm this prediction of the two-layer model of integration by demonstrating that there is a lower threshold for evoking a somatic action potential when excitation is directed on a single dendrite than when it is directed towards multiple dendrites (Figure 7A; AP threshold for Oblique: multiple = 5.4 ± 0.09 µJ, single = 3.84 ± 0.08 µJ, *n* = 8, *P* < 0.05, paired *t*-test). Interestingly, there is little difference in terms of the action potential threshold at the tuft (Figure 7B; AP threshold for Tuft: single = 8.04 ± 0.18 µJ, multiple = 8.82 ± 0.09 µJ, *n* = 5, *P* > 0.1, paired *t*-test).

We next examined and compared the kinetics of the responses to single- and multibranch excitation at each of the three domains of the pyramidal neuron (Figure 8A). Studies were first carried out on individual branches from the three dendritic domains of the same cell (left, Figure 8A). For photolytically induced depolarizations at the tuft that were >5–10 mV the durations of the responses at half maximum were largely >100 ms for both single- and multi-branch excitation (Figures 6–8). In contrast to the results at the tuft, the duration of depolarizations at the oblique and basal dendrites to strong stimuli were typically longer for single branch excitation (right, Figure 8A; Figures 2 and 5). Thus, at stimulus evoked depolarizations above the expected threshold for evoking a somatic action potential, the onset and duration of the multibranch oblique and basal responses remained fast and brief. Because of variable activation of repolarizing conductances a more reliable measure of response kinetics is the time to peak depolarization (Figure 8A, right panel; One oblique: 50.81 ± 3.37 ms, *n* = 20; One basal: 54.69 +/− 4.9 ms, *n* = 9; One tuft: 66.24 ± 4.24 ms, *n* = 13; Multiple oblique: 23.69 ± 1.27 ms, *n* = 15; Multiple basal: 25.96 ± 3.36 ms, *n* = 5; Multiple tuft: 50.09 ± 3.30 ms, *n* = 10).

### Functional advantage of multibranch integration

The tradeoff between response efficiency and kinetics for oblique dendrites predicts that multibranch integration of the oblique dendrites could enable precise entrainment at higher frequencies. In addition, the absence of fast multibranch integration at the tuft suggests that the tuft would not be able to support entrainment at similar high frequencies. We first tested these predictions in the absence of TTX by directing trains of five stimuli at different frequencies at tufts (Figure 8B). The stimulus intensity was set at a level that reliably elicits an action potential. The five stimuli were then given at progressively faster frequencies. The highest frequency at which the somatic action potential could still precisely follow the dendritic input (entrainment) was recorded (low trace of each pair of traces in Figure 8B). For multiple oblique inputs the entrainment frequency was significantly higher than for tuft inputs (16.7 ± 3.8 vs. 5.7 ± 1.3 Hz, respectively; paired *t*-test, *p* < 0.05). Precise entrainment by multibranch oblique integration was also significantly faster than for single branch integration (16.7 ± 3.8 vs. 2.8 ± 0.4 Hz, *p* < 0.05). We next tested these predictions using electrical stimulation of their respective excitatory pathways in the presence of GABA_A_ and GABA_B_ receptor antagonists (10 µM SR 95531 and 10 µM CGP 35348 respectively). Electrical stimulation at intensities strong enough to reliably evoke action potentials it is likely to activate multiple dendritic branches. Consistent with the photostimulation responses, multibranch electrical stimulation of the oblique dendrites led to precise entrainment over a wider frequency range than tuft entrainment. (Figure 8C; SC stimulation: 4.9 ± 0.7 Hz; PP stimulation: 2.6 ± 0.5 Hz; paired *t*-test, *n* = 6, *p* < 0.05).

## Discussion

This study compared the properties of single- and multibranch integration to complex patterns of photostimulation as a means to probe and compare the global and the two-stage model of dendritic integration. This strategy provided the means to implement non-linear and linear integration at precise locations using clustered and distributed inputs. Non-linear integration is an integral part of both modes of integration. The critical difference lies in their loci of non-linear integration and the active conductances that are recruited at those loci. The results suggest that both global and two-stage integration can drive somatic outputs. Novel findings reported here include the significant differences in the kinetics of dendritic integration response between single- and multibranch integration at the oblique and basal dendrites. Single branch integration possesses a low threshold for evoking a fast sodium- and slow NMDAR-mediated local dendritic spike compared to multibranch integration. But this increased sensitivity is achieved at a cost of response kinetics. This tradeoff between sensitivity and integration kinetics is further confirmed by the finding of significant differences in spike entrainment. Indeed, multibranch integration can precisely entrain somatic action potentials 6-fold faster than single branch integration. It is important to note that the maximal entrainment frequency described here is not the same as the maximal frequency. In fact, the low entrainment frequency to single branch integration is due to the production of additional spikes. It is as if single branch integration is better suited for eliciting bursting responses. This would not be surprising since the degree of recruitment of voltage gated calcium conductances responsible for burst firing increases with the duration of depolarization (Kay and Wong, 1987). Why is this finding significant? It is relevant for an ongoing controversy on a fundamental issue in neuroscience, what is the format of the information that is transmitted in the brain. Is the information being transmitted through “spike timing” or “spike rate” (or the number of spikes)? The findings here suggest that multibranch integration would better preserve the information in spike timing transmission, whereas single branch integration would best optimize information being transmitted via spike rate. Single branch integration provides the input-output transfer function with high dynamic range. The latter would also be well suited for initiating burst firing modes. Our findings do not weigh in on the spike timing vs. spike rate controversy. But they suggest that dendrites of pyramidal neurons have the capacity to support both mode of signal transmission.

A second consistent observation from this study is that the behavior of multibranch integration is domain-specific. Multibranch integration of oblique dendrites is sublinear, whereas multibranch integration of tuft dendrites is supralinear at moderate stimulation intensities. This dichotomy exists even though the response of individual tuft and oblique dendrites are qualitatively indistinguishable. Furthermore, this dichotomy extends to their ability for precise spike entrainment. These observations are not simply a phenomena related to the photolysis technique, since the differences in entrainment were also apparent to synaptic stimulation. The mechanistic basis for the domain specific integration is likely to lie in the expression of different voltage-dependent conductances at the differing loci where distal dendrites converge. At the base of the tuft where the distal tuft branches converge, voltage gated calcium channels are expressed in high densities (Larkum et al., 1999, 2009). The sublinear summation observed for multibranch oblique integration suggests that the recruitment of the A-type potassium conductance at the proximal apical trunk outweighs the recruitment of voltage-gated calcium and NMDA conductances. The preferential recruitment of the potassium conductance with multibranch oblique integration can be explained simply by the fact that NMDA receptors in the region of dendritic convergence on the apical trunk are not exposed to glutamate, yet the A-type potassium conductances can be activated by distant excitation. This study does not compare the relative expression the A-type conductance on the main apical trunk and on the thin oblique dendrites. On the distal dendrites NMDA and the A-type potassium conductances may be well counter-balanced (Cash and Yuste, 1999; Gasparini and Magee, 2006; Losonczy and Magee, 2006; Losonczy et al., 2008). The differential expression of conductances at the base of the tuft and the proximal apical trunk suggests that the tuft and the oblique dendrites may employ different temporal coding strategies. However, predicting in vivo behavior from in vitro observations must always be done with caution since it is difficult to account for the many presynaptic factors such as feedforward and feedback inhibition that contribute to in vivo behavior.

This study provides an experimental demonstration of a widely held belief that the dendritic arbor can support non-linear integration at multiple locations (Mel, 1993; Schiller et al., 2000; Wei et al., 2001; Polsky et al., 2004; Gasparini and Magee, 2006; Losonczy and Magee, 2006; Johnston and Narayanan, 2008; Major et al., 2008; Larkum et al., 2009; Branco and Hausser, 2011). However, this is the first study to systematically examine the kinetic consequences of supralinear integration at different locations on the dendritic arbor and to demonstrate the tradeoffs between temporal precision and signal amplification. There is no single mode of integration with optimal performance. But the ability to switch between different loci of non-linear integration would provide the flexibility to optimize response to a specific condition. The findings here may be relevant to the controversy on whether information is transmitted in the form of spike timing or spike rate and the contributory role of dendritic integration.

## Conflict of interest statement

The authors declare that the research was conducted in the absence of any commercial or financial relationships that could be construed as a potential conflict of interest.
